# Assessing the cardioprotective effects of exercise in APOE mouse models using deep learning and photon-counting micro-CT

**DOI:** 10.1371/journal.pone.0320892

**Published:** 2025-04-10

**Authors:** Alex J. Allphin, Rohan Nadkarni, Zay Y. Han, Darin P. Clark, Ketan B. Ghaghada, Alexandra Badea, Cristian T. Badea

**Affiliations:** 1 Quantitative Imaging and Analysis Lab, Department of Radiology, Duke University Medical Center, Durham, North Carolina, United States of America; 2 Department of Radiology, Baylor College of Medicine, Houston, Texas, United States of America; 3 Department of Radiology, Texas Children’s Hospital, Houston, Texas, United States of America; George Emil Palade University of Medicine Pharmacy Science and Technology of Targu Mures: Universitatea de Medicina Farmacie Stiinte si Tehnologie George Emil Palade din Targu Mures, ROMANIA

## Abstract

**Background:**

The allelic variations of the apolipoprotein E (APOE) gene play a critical role in regulating lipid metabolism and significantly impact cardiovascular disease risk (CVD). This study aimed to evaluate the impact of exercise on cardiac structure and function in mouse models expressing different APOE genotypes using photon-counting computed tomography (PCCT) and deep learning-based segmentation.

**Methods:**

A total of 140 mice were grouped based on APOE genotype (APOE2, APOE3, APOE4), sex, and exercise regimen. All mice were maintained on a controlled diet to isolate the effects of exercise. Low dose cardiac photon counting micro-CT imaging with intrinsic gating was performed using a custom-built micro-PCCT system and data was reconstructed with an iterative algorithm incorporating both temporal and spectral dimensions. A liposomal-iodine nanoparticle contrast agent was intravenously administered to uniformly opacify cardiovascular structures. Cardiac structures were segmented using a 3D U-Net deep learning model that was trained and validated on manually labeled data. Statistical analyses, including ANOVA, post-hoc analysis, and stratified group comparisons, were used to assess the effects of genotype, sex, and exercise on key cardiac metrics, including ejection fraction and cardiac index.

**Results:**

The PCCT imaging pipeline provided high-resolution images with enhanced contrast between blood compartment and myocardium allowing for precise segmentation of cardiac features. Deep learning-based segmentation achieved high accuracy with an average Dice coefficient of 0.85. Exercise significantly improved cardiac performance, with ejection fraction increasing by up to 18% and cardiac index by 46% in exercised males, who generally benefited more from exercise. Females, particularly those with the APOE4 genotype, also showed improvements, with a 31% higher ejection fraction in exercised versus non-exercised mice. Stratified analyses confirmed that both sexes benefited from exercise, with males showing larger effect sizes. APOE3 and APOE4 genotypes derived the greatest benefit, while APOE2 mice showed no significant improvement.

**Conclusions:**

This study demonstrates the utility of PCCT combined with deep learning segmentation in assessing the cardioprotective effects of exercise in APOE mouse models. These findings highlight the importance of genotype-specific approaches in understanding and potentially mitigating the impact of CVD through lifestyle interventions such as exercise.

## Introduction

Cardiovascular disease (CVD) and Alzheimer’s disease (AD) risk are associated with aging, and modifiable lifestyle factors such as sedentary behavior and diets high in fats and sugars. These diseases are further influenced by genetic factors, notably the apolipoprotein E (APOE) gene, which plays a crucial role in lipid metabolism [1–3]. Understanding the interplay between genetic predisposition, environmental factors, and lifestyle choices such as exercise is critical for developing effective therapeutic and preventive interventions. Among the various imaging modalities, x-ray computed tomography (CT)-based quantitative imaging stands out for its ability to produce high-resolution, anatomical and functional multi-dimensional images, which are crucial for studying disease mechanisms and evaluating therapeutic interventions. In particular, photon-counting CT (PCCT) represents a significant advancement in imaging technology, offering superior image quality and enhanced quantitative capabilities compared to traditional CT methods. PCCT’s benefits include higher spatial resolution, improved tissue contrast, and reduced radiation dose, making it a promising tool for both preclinical and clinical applications [4]. Photon-counting CT has demonstrated promising results in cardiovascular imaging, particularly for detecting coronary calcifications [5], assessing stents [6], and evaluating myocardial perfusion [7]. The technology’s ability to reduce electronic noise and artifacts, combined with its multi-energy capabilities, allows for superior tissue characterization, making it highly suitable for complex cardiac imaging tasks.

Preclinical research, especially in mouse models, is an essential precursor and companion to clinical research because it allows precise control over genetic and environmental variables [8]. Our group has previously demonstrated the value of preclinical PCCT imaging in cancer and cardiac studies in mice, emphasizing its potential for detailed phenotypic characterization [9,10]. Despite these advances, the application of PCCT in preclinical cardiac imaging, particularly for evaluating the impact of exercise as a therapeutic intervention, remains underexplored and warrants further investigation.

Previous preclinical research has focused on quantitatively comparing the cardiac anatomy and function of different APOE mouse models using only 3D left ventricle segmentations [11,12]. This left ventricle segmentation has enabled calculation of key cardiac metrics such as stroke volume, ejection fraction, and cardiac output, providing a quantitative representation of cardiac health. The different APOE genotypes used in these studies served as key models for varying degrees of risk for Alzheimer’s disease and cardiovascular disease, offering valuable insights into the genetic predisposition and progression of these conditions [1–3].

In our previous study, we developed a cardiac photon-counting CT (PCCT) pipeline to phenotype APOE mouse models and investigated the effects of high fat diet on cardiac performance across different APOE genotypes [12]. In the current study, we expanded upon our previous work by incorporating additional cardiac metrics derived from a larger set of cardiac features and by introducing exercise as an experimental variable. While exercise is known to provide significant cardiovascular benefits, its effects across different APOE genotypes remain poorly understood. To address this, we employed a deep learning-based approach for full heart segmentation, which offers greater speed, reliability, and effectiveness compared to previously used atlas-based methods limited by their reliance on predefined anatomical templates and manual corrections, leading to slower processing times and potential inaccuracies [13]. By utilizing these advanced techniques, we aim to deliver a more detailed and comprehensive assessment of the benefits of exercise in APOE mouse models genetically predisposed to cardiovascular disease.

## Methods

### Cardiac imaging pipeline

**Fig 1:**
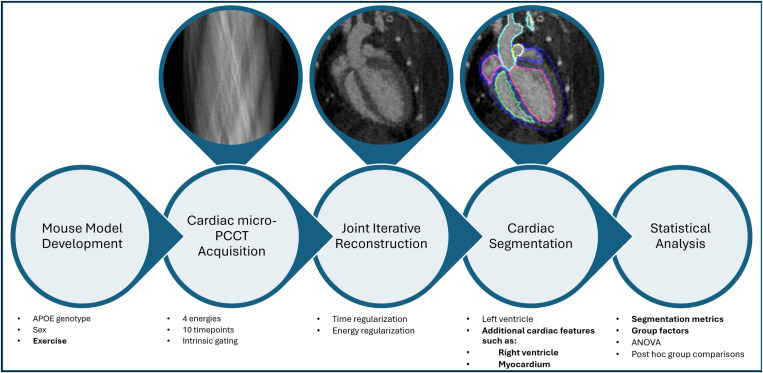
Diagram showing the steps in our cardiac imaging pipeline. We have bold-faced the items that relate to new aspects unique to this work (compared to our previous study [12]).

Fig 1 illustrates the cardiac photon-counting CT (PCCT) pipeline developed in our previous work [12] and highlights the modifications introduced in the current study [12].

The pipeline integrates advanced imaging and deep learning techniques to quantitatively assess cardiac function. The core imaging methods, including contrast-enhanced cine-PCCT, intrinsic cardiac gating, and multi-energy iterative reconstruction, were adapted from our previous work [12] to additionally evaluate exercise as a group factor.

Imaging was conducted using our custom-built micro-PCCT system [14] at the Duke Quantitative Imaging and Analysis Lab. Each of the imaging datasets was reconstructed using a 5D joint iterative reconstruction algorithm, incorporating regularization along both time and energy dimensions as described previously [10,12,15]. The resulting PCCT data were then used for deep learning-based cardiac segmentation and statistical analysis. Detailed explanations of these techniques are provided in the following sections.

### Mouse models

All animal procedures were approved by the Duke Institutional Animal Care and Use Committee (IACUC, protocol registry number: A173-20–08). The study utilized a cohort of 140 mice bred and maintained at Duke University Medical Center’s facilities in the Bryan Research Building for Neurobiology. At the end of the study, the mice were euthanized using an intraperitoneal injection of 250 mg/Kg pentobarbital, as approved by our institution’s animal care and use committee. We ensured that all actions were carried out humanely, and with the utmost regard for the welfare of our animals. The experimental design incorporated variation in APOE genotype (APOE2, APOE3, APOE4), sex (male, female), and exercise regimen. We also used some APOE mouse models that possess a component of humanized innate immune system through the inclusion of the human (h)NOS2 gene as described in previous work [12]. The presence or absence of this humanized component is abbreviated as “HN factor” throughout this work. The average age of all animals was 14.5 months with a standard deviation of 3.3 months. All animals were maintained on the same normal diet to eliminate dietary effects, allowing us to isolate the impact of exercise. The exercised mice were provided with running wheels for 1 hour per day, 5 days a week, over a period of 3 months. Each mouse was housed individually during the designated time, ensuring unrestricted access to the wheel. Exercise activity was monitored using the Wheel Manager software (Med Associates Inc., St. Albans, VT, USA), and the distance run was recorded at the end of each session. Table 1 details the distribution of mice across these experimental variables, and Table 2 provides percentages of exercised mice, and HN factor within each genotype. While the distribution of animals across genotypes and sexes appears relatively balanced in terms of total numbers, there is some variability in exercise participation within each group, particularly when broken down by sex.

**Table 1:** Summary of cohort statistics grouped by genotype, sex, and exercise participation.

Genotype	Sex	Exercise?	Count
APOE2	Female	Yes	20
		No	7
	Male	Yes	18
		No	9
APOE3	Female	Yes	15
		No	8
	Male	Yes	16
		No	6
APOE4	Female	Yes	12
		No	7
	Male	Yes	12
		No	10

**Table 2:** Overview of experimental group composition with percentages of the HN and exercise factors in each genotype. This table has been included primarily to communicate the relative counts for each experimental factor (i.e., sex, HN factor, and exercise).

Genotype	Male	Female	%HN	%Exercise
APOE2	27	27	42.6%	70.3%
APOE3	22	23	48.8%	68.9%
APOE4	22	19	48.8%	58.5%

### *In Vivo* PCCT imaging

Mice were imaged using our custom PCCT system, which features a Varian G297 x-ray tube and a Santis 1604 PCD (Dectris, Inc.), configured with 4 energy bins and a CdTe sensor [14]. The system provides a field of view of 12.9 cm x 4.3 cm and captures high-resolution images with a pixel size of 150 µm. During imaging, mice were anesthetized with 2–3% isoflurane delivered via a nosecone, and vital signs were monitored throughout the procedure using a pneumatic pillow for respiration and electrodes for ECG. To enhance blood-pool contrast, long-circulation liposomal iodine (Lip-I) nanoparticle contrast agent was administered via retro-orbital injection. A volume of 0.012 mL (equaling 1.2 mg of I [9,16]) was injected per gram of body weight for each mouse. Scanning parameters included an 80 kVp tube voltage, 4 mA tube current, and 10 ms/exposure, resulting in a total scan time of 70 seconds and an absorbed radiation dose of approximately 118 mGy. This dose is 55 to 76 times less than the lethal dose of 6.5–9 Gy, known as LD50/30 [17]. The PCD energy thresholds were set at 25, 34, 50, and 60 keV, with the second energy threshold positioned near the K-edge of iodine (33.2 keV) to optimize iodine contrast enhancement. As in our previous studies, a semi-automated intrinsic gating approach [12] was employed to sort the projections into 10 cardiac phases during the R-R interval of the cardiac cycle, enabling high-fidelity assessment of cardiac function.

### 5D CT reconstruction and material decomposition

Reconstruction of the PCCT data was performed using a multi-channel iterative algorithm with an isotropic voxel size of 125 µm. The reconstruction process involved joint regularization across both the temporal and spectral dimensions using rank-sparse kernel regression (RSKR) [15,18].

Reconstruction iteratively computed Xthat is the solution to the following equation:


$$\begin{array}{c} \hat{X}=\underset{X}{\arg\min}\frac{1}{2}\underset{t}{\sum }\underset{e}{\sum }\left[{\left|\left|RX\left(t,e\right)-Y\left(t,e\right)\right|\right|}_{2}^{2}+\text{λ}\left(t,e\right)\text{Reg}\left(\text{X}\right)\right] \end{array}$$ (1)

The reconstructed data (columns of X) at each energy (e) and time (t) minimizes the reprojection error (R representing the system projection matrix) relative to log-transformed projection data (Y). Projections are temporally selected based on the intrinsic gating procedure previously discussed. To reduce noise in the reconstructed results, the data regularizer ($\text{Reg}\left(�\right)$) RSKR reduces bilateral total variation (BTV) jointly across the spectral dimension and patch-based rank across the time dimension.

Reconstruction resulted in 5D volumes with 4 energies, 10 cardiac phases, and 3 spatial dimensions with 125 µm isotropic voxels. Iterative reconstruction times ranged from 5–9 hours depending on the volume size.

Since our reconstructed volumes have 4 energies, we could use material decomposition to generate I, photoelectric effect (PE), and Compton scattering (CS) maps. Material decomposition was performed using a variation of the method described by Alvarez and Macovski [18,19].

In essence, material decomposition was performed via matrix inversion, solving the following linear system at each voxel:


$$\begin{array}{c} QUOTE \end{array}$$ (2)

In this equation, X is the reconstructed PCCT image vectorized as columns by energy, C represents the concentrations of our basis materials (e.g., I, PE and CS or I, Ca, and H_2_O) for each voxel, and M is a matrix of material sensitivities at each energy. An orthogonal subspace projection approach was used to prevent negative concentrations [18]. Post-decomposition, the material maps were assigned colors and combined in ImageJ for visualization.

These material maps were used to distinguish between blood (containing iodine contrast agent) and other structures such as soft tissue and bone, potentially facilitating a more precise analysis of cardiac features.

### Deep learning-based cardiac segmentation

The goal of quantitative imaging is to extract useful, interpretable, and repeatable markers of biological performance. To quantitatively assess cardiac anatomy and function using micro-PCCT, we used segmentations of cardiac features at ventricular diastole and systole to calculate metrics such as ejection fraction and cardiac output. In this study, we expanded our previous approach which used segmentation of the left ventricle to further include segmentations of both ventricles, both atria, the myocardium, and several peripheral vessels, including the pulmonary artery and aorta. High-throughput imaging requires a robust and rapid segmentation approach, making deep learning an ideal choice. Consequently, we trained a deep learning-based segmentation network using an initial subset of user-labeled data.

These training labels were created using the seed-growing and painting tools in 3D Slicer [20]. Initially, seeds were placed in each of the cardiac structures, which were then grown to generate relatively accurate segmentations [21]. Finally, the labelers manually refined the segmentations using the 3D painting tools. A total of 46 sets of manual labels were created to train and validate the segmentation network. The creation of these labels was a collaborative effort among four researchers, each with at least two years of cardiac imaging experience. Involving multiple individuals in label creation helped increase throughput and mitigate the risk of the network learning a specific individual’s bias, given the user discretion involved in both seed placement and refinement.

For this segmentation task, we selected the 3D UNet CNN architecture [22], adapted from previous work [23]. We trained two separate networks using different input data types to assess potential differential benefits. For one network, the input was a cropped (128³ voxels) 3D view of the lowest energy threshold CT image. For the other network, the input was the same cropped region but from the decomposed iodine map. The 46 manual labels (comprising both diastole and systole of 23 mice) were used for training. These labels were randomly shuffled, independent of phase, and split into dedicated training (36 segmentations), validation (5 segmentations), and test (5 segmentations) sets. To maximize network generalizability and avoid overfitting, we employed multiple data augmentation strategies, including random cropping, random rotation, and random intensity shifts. We trained the models for 200 epochs at a learning rate of 0.001 using the Adam optimizer [24]. This training process took approximately two hours on a single RTX 5000 GPU. We used cross-entropy loss as the training cost function and evaluated network performance on the dedicated test set by calculating voxel-by-voxel accuracy, Jaccard index, Dice coefficient, precision, and recall. As shown later in the results section, due to its superior performance, the CT-input model was used for cardiac metric calculation and statistical analysis.

### Derived cardiac metrics

Using the segmentations, we calculated the following key cardiac metrics: stroke volume (SV), ejection fraction (EF), cardiac output (CO), cardiac index (CI), and myocardial mass (MM). Equations 3–6 show how these metrics were calculated. The heart rate (HR), used in Equation 5, was measured during the intrinsic gating procedure and thus represents the anesthetized heart rate. The mouse body mass (m) used in Equation 6 was measured using a digital scale at the start of the day in which imaging occurred.

SV was calculated as the difference between end-diastolic volume (EDV) and end-systolic volume (ESV):


$$\begin{array}{c} SV=EDV-ESV \end{array}$$ (3)

EF, representing the percentage of blood ejected from the left ventricle, was calculated as:


$$\begin{array}{c} EF\left(\%\right)=\frac{SV}{EDV}\times 100 \end{array}$$ (4)

CO, representing the volume of blood pumped per minute, was calculated as:


$$\begin{array}{c} CO=SV\times HR \end{array}$$ (5)

CI which represents the weight-normalized version of CO, was calculated as:


$$\begin{array}{c} CI=\frac{CO}{m} \end{array}$$ (6)

MM was approximated using the average volume of the segmented myocardium across both the diastolic and systolic phases. This myocardial volume was converted to MM using an assumed tissue density of 1.053 g/mL [25].

By default, the EDV and ESV were derived from the segmentation of the left ventricle. We have also included some metrics that were calculated using the right ventricle segmentation. In these cases, we have added the prefix “RV” to indicate this distinction. For example, EF is the ejection fraction calculated from the left ventricle; RVEF is the ejection fraction calculated from the right ventricle.

### Multi-factor statistical analysis

We first assessed the normality of each cardiac metric using the Shapiro-Wilk test [26]. Homogeneity of variance was checked using Levene’s test to evaluate whether variances were equal across groups [27]. Three metrics (mass, ejection fraction and myocardial mass) did not satisfy the normality test. Mass did not satisfy either test.

For metrics that passed the normality and homogeneity tests, we applied multi-factor ANOVA to evaluate the main effects and interactions of exercise, sex, genotype, and HN factor. More explicitly, we used linear models for each cardiac measure (e.g., Cardiac output ~ Genotype*Exercise*Sex*HN), where Genotype represents APOE2, APOE3, and APOE4, Exercise indicates exercised or non-exercised groups, and HN is 0 for mouse lines with mNos2 background and 1 for those with mNos2-/- hNOS. These results are presented both numerically and visually, providing a clear understanding of the genotype, exercise, sex, and HN factor interaction effects on cardiac performance. We applied the Benjamini-Hochberg procedure for False Discovery Rate (FDR) correction to adjust p-values for multiple comparisons [28]. The effect size was measured using eta-squared [29] to estimate the proportion of variance explained by each factor.

Metrics that failed the normality and/or homogeneity of variance tests were analyzed using other applicable methods. For mass, which failed both assumptions, we used the Kruskal-Wallis test [30] with Dunn’s post hoc test [31] for basic multifactor comparisons. For ejection fraction and myocardial mass, which failed only the normality assumption, we used generalized linear models (GLMs) for full analysis of factors and their interactions. Specifically for the metrics in this work, we employed GLMs with the Gamma distribution family and log link function, as recommended for skewed continuous data [32].

### Stratified subgroup exercise comparison

We also performed a series of stratified group comparisons to examine the influence of exercise within specific subgroups, such as sex and genotype, using the Mann-Whitney U test for non-parametric comparisons. This approach was chosen to account for potential violations of normality in smaller subgroups. For example, we compared exercised and non-exercised male mice separately. We extended these stratified comparisons across combinations of sex and genotype to explore how exercise manifests differently in these subgroups. We once again applied FDR corrections to all p-values in these stratified group comparisons to reduce the likelihood of Type I errors. All analyses were performed using Python including the *statsmodels* and *scipy* packages. A significance threshold of 0.05 was used for all statistical tests.

## Results

### Segmentation results

**Fig 2:**
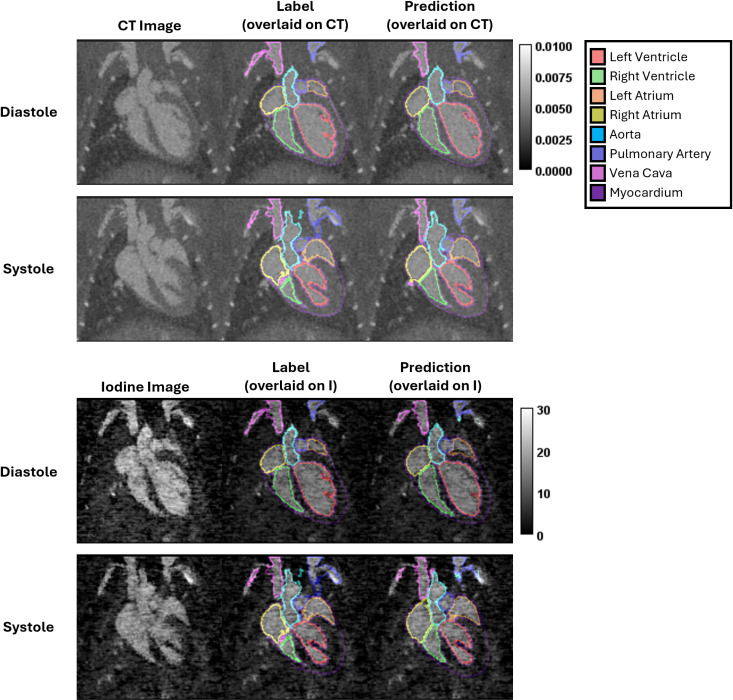
Test set qualitative segmentation performance overview. Within the images labelled “Label” and “Prediction,” the outline of each segmentation is shown as an overlay on either a CT image (upper half of figure) or a decomposed I image (bottom half of figure). The CT images are shown as attenuation maps with units of 1/voxel size while the I images represent concentrations with units of mg/mL. Each segmentation color represents a different anatomical region. We reiterate that the segmentations are voxelated 3D semantic segmentations; we show only the 2D outline of the segmentations in these images for visual simplicity.

Table 3 contains the segmentation performance metrics calculated on the test set. We have included calculations of accuracy, Jaccard index, Dice coefficient, precision, and recall for each anatomical segment as well as an average across all segments. For example, the average Dice coefficient across all segmented structures when trained using CT images was 0.85 which is considered acceptable for most medical images. We note that the segmentation performance for CT images is better than for I maps (average Dice: 0.72). This is most likely due to the higher noise in I images which leads to increased uncertainty along feature boundaries. We also note that the left ventricle has the lowest overlap scores (Dice and Jaccard) likely due to the ambiguity present in separating the aorta from the left ventricle. That region required the most discretion on the part of the label creator. Theoretically the network prediction represents an unbiased result, but further work would be required to verify that claim. Figure 2 shows a representative example indicating qualitative segmentation performance.

**Table 3:** Test set quantitative segmentation performance summary. The upper half of the table shows the performance of the network when trained using CT images as input while the bottom half shows performance when trained with I maps as input. We show mean values of accuracy, Jaccard index, Dice coefficient, precision, and recall for each anatomical segment. We also show an overall mean value for each performance metric across all segments.

	Segment Name	Accuracy	Jaccard	Dice	Precision	Recall
**CT Input**	LV	0.9979	0.6635	0.7772	0.9844	0.6671
	RV	0.9988	0.8047	0.8886	0.9235	0.8566
	LA	0.9991	0.7703	0.8641	0.9004	0.8335
	RA	0.9986	0.8441	0.9137	0.9472	0.8837
	Aorta	0.9965	0.7551	0.8538	0.8828	0.8321
	Venae Cavae	0.9973	0.6738	0.7936	0.8361	0.8058
	Pulmonary Artery	0.9917	0.7336	0.8404	0.8596	0.8259
	Myocardium	0.9904	0.7856	0.8757	0.8283	0.9324
	Mean	**0.9963**	**0.7538**	**0.8509**	**0.8953**	**0.8296**
	Standard Deviation	**0.0034**	**0.0621**	**0.0463**	**0.0546**	**0.0767**
**Iodine Input**	LV	0.9966	0.5313	0.6843	0.8866	0.5782
	RV	0.9970	0.6166	0.7607	0.7890	0.7473
	LA	0.9973	0.5668	0.7214	0.6464	0.8219
	RA	0.9973	0.7196	0.8356	0.8229	0.8615
	Aorta	0.9932	0.5586	0.7134	0.7340	0.7031
	Venae Cavae	0.9956	0.4726	0.6395	0.5641	0.7964
	Pulmonary Artery	0.9851	0.5244	0.6824	0.6813	0.6967
	Myocardium	0.9810	0.6175	0.7623	0.7541	0.7782
	Mean	**0.9929**	**0.5759**	**0.7249**	**0.7348**	**0.7479**
	Standard Deviation	**0.0063**	**0.0753**	**0.0607**	**0.1028**	**0.0887**

### Overview of cardiac metrics

Table 4 provides the average and standard deviation of the cardiac metrics, categorized by sex, genotype, and exercise factor. Values are presented as means with standard deviations in parentheses. The raw volumetric measurements for each heart chamber can be found in the supplemental S1 Table 1.

**Table 4:** Averages and standard deviations for quantitative metrics grouped by sex, genotype, and exercise state. Standard deviations are shown in parentheses below each average value. From left to right the quantitative metrics are mass, heart rate (HR), stroke volume (SV), ejection fraction (EF), cardiac output (CO), cardiac index (CI), right ventricular stroke volume (RVSV), right ventricular ejection fraction (RVEF), and myocardial mass (MM).

Sex	Genotype	Exercise?	Mass (g)	HR (bpm)	SV (mL)	EF (%)	CO (mL/min)	CI (mL/min/g)	RVSV (mL)	RVEF (%)	MM (mg)
Male	APOE2	Yes	31.467 (1.439)	468.571 (47.003)	0.033 (0.008)	55.104 (6.379)	15.253 (3.840)	0.484 (0.117)	0.030 (0.007)	0.484 (0.077)	197.4 (42.2)
		No	31.678 (1.707)	470.857 (48.618)	0.026 (0.006)	47.106 (11.716)	12.176 (3.856)	0.382 (0.112)	0.025 (0.007)	0.416 (0.117)	203.1 (35.2)
	APOE3	Yes	34.131 (1.826)	464.786 (45.435)	0.033 (0.005)	51.988 (5.775)	15.268 (3.175)	0.448 (0.090)	0.030 (0.005)	0.447 (0.089)	197.4 (22.9)
		No	33.750 (5.381)	430.429 (15.452)	0.023 (0.002)	39.746 (8.566)	10.058 (1.367)	0.307 (0.072)	0.021 (0.002)	0.354 (0.049)	188.4 (27.3)
	APOE4	Yes	30.750 (2.248)	483.286 (25.255)	0.033 (0.007)	52.396 (7.984)	15.666 (3.085)	0.509 (0.093)	0.029 (0.005)	0.499 (0.059)	202.2 (36.8)
		No	26.640 (5.371)	448.714 (26.367)	0.020 (0.004)	44.511 (7.540)	8.938 (1.627)	0.348 (0.094)	0.018 (0.004)	0.379 (0.075)	151.8 (27.9)
Female	APOE2	Yes	26.510 (1.981)	449.743 (48.957)	0.028 (0.005)	56.147 (6.017)	12.547 (2.863)	0.474 (0.105)	0.025 (0.004)	0.494 (0.062)	157.3 (21.2)
		No	25.929 (4.555)	464.571 (18.582)	0.025 (0.005)	60.886 (6.161)	11.782 (2.314)	0.457 (0.066)	0.023 (0.005)	0.488 (0.022)	141.3 (16.8)
	APOE3	Yes	27.887 (2.244)	470.800 (42.496)	0.024 (0.007)	52.783 (13.221)	11.510 (3.286)	0.412 (0.113)	0.023 (0.008)	0.481 (0.100)	153.8 (31.1)
		No	29.438 (1.586)	448.821 (28.004)	0.021 (0.005)	45.652 (10.075)	9.291 (2.433)	0.317 (0.092)	0.018 (0.005)	0.406 (0.130)	156.8 (15.1)
	APOE4	Yes	26.267 (5.062)	489.500 (27.180)	0.024 (0.003)	58.190 (5.623)	11.722 (1.642)	0.455 (0.066)	0.022 (0.003)	0.537 (0.060)	150.9 (16.9)
		No	28.400 (2.772)	455.755 (51.694)	0.018 (0.006)	44.509 (13.259)	8.330 (3.413)	0.296 (0.128)	0.019 (0.005)	0.463 (0.152)	154.2 (15.0)

Table 4 offers a general overview of the sample measurements of key cardiac metrics within our cohort grouped by sex, genotype, and exercise. Most relevant to this work, we measured differences between the mean values of exercised mice and nonexercised mice within some sex and genotype groups. For example, exercised mean EF values were 31% higher in APOE3 males, 18% higher in APOE4 males, 16% higher in APOE3 females, and 31% higher in APOE4 females. Similarly, exercised mean SV values were 27% higher in APOE2 males, 44% in APOE3 males, and 65% higher in APOE4 males, but 14% in APOE3 females and 33% in APOE4 female. Mean CO values also improved with exercised CO values being 51% higher in APOE3 males and 75% higher in APOE4 males, compared to 24% in APOE3 females and 41% in APOE4 females. This suggests a possible sex- and genotype-specific improvement in the heart’s ability to pump blood.

Genotype-specific responses showed that APOE4 mice had the most pronounced improvements in cardiac function. Exercised mean CO values were 75% higher in APOE4 males and 30% higher in females from the same genotype highlighting a strong cardioprotective effect of exercise in this genotype. APOE2 mice exhibit more modest improvements with 27% higher mean SV values in males and 12% higher mean SV values females suggesting a weaker but still positive response to exercise.

Sex differences were apparent, with males generally having higher baseline CO and MM compared to females. Despite this, exercise improved cardiac efficiency, measured as mean CI, in both sexes. APOE4 males showed a 46% improvement in CI, while APOE4 females displayed a similar 44% improvement, demonstrating that the benefits of exercise are not exclusive to one sex.

Exercise had to have little to no impact on body mass or MM. These findings suggest that exercise predominantly enhanced cardiac function rather than inducing changes in size or structure.

To summarize, within our cohort, the clearest measured differences were in the cardiac performance metric between exercised and nonexercised mice. These differences were most pronounced in APOE4 females, APOE4 males, and APOE3 males. However, statistical analysis presented in the following section is required to understand the significance of these differences.

### Multi-factor statistical results

**Fig 3:**
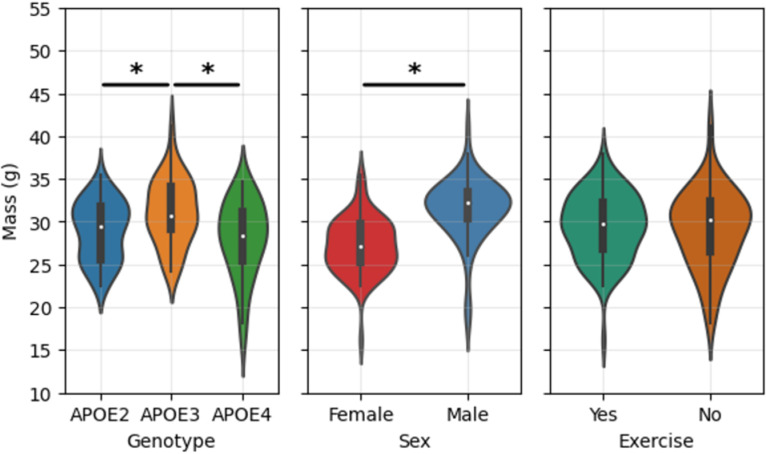
Violin plots of mass across genotype, sex, and exercise groups. The lines with asterisks indicate significant differences (p<0.05) identified by the Kruskal-Wallis test and (when needed) Dunn’s post hoc test. These plots coupled with other findings indicate that mass differences in our cohort can be attributed to sex and genotype but not **to** exercise.

Mass failed both the normality and homogeneity of variance assumptions and was therefore analyzed using the Kruskal-Wallis and Dunn’s tests. As shown in Fig 3, significant mass differences were observed in our cohort between genotype levels and between sexes but notably not between exercise groups.

EF and MM each failed the assumption of normality and were analyzed using GLMs. For EF, no main effects were found to be significant but there were significant interactions between genotype and exercise as well as sex and exercise. Specifically, APOE4 mice without exercise showed a significant (p=0.014) reduction in EF compared to baseline. Additionally, males without exercise also showed a significant (p=0.017) reduction in EF. For MM, sex was found to be a highly significant (p<0.0001) main effect with males showing an overall larger MM than females. Interestingly, the interaction term between sex and HN factor was significant (p=0.009), demonstrating a potential difference in how the human Nos2 gene effects male and female mice. Specifically, males with the HN factor showed a decrease in MM.

All other metrics outside of mass, EF, and MM passed the relevant assumptions and were analyzed as part of a multi-factor ANOVA. As shown in Table 5, exercise was a significant predictor with very large effect sizes for nearly all key cardiac metrics including SV, CO, CI, RVSV, and RVEF. Sex was also a significant predictor for almost all cardiac metrics; however, CI was an exception. This indicates that some sex differences, such as those for CO, may be attributed to sex differences in body mass rather than sex differences in exercise response. Genotype was shown to be a significant predictor with a small to medium effect size for SV and CI. The exercise-sex interaction was also significant with small to medium effect sizes for SV and CO.

**Table 5:** A summary of all significant findings from the multi-factor ANOVA. For brevity, we have included only the metrics and effects that were statistically significant (p<0.05). In the final column, we have provided a basic interpretation of those results. Effect sizes are described relatively with the values 0.01, 0.06, and 0.14 representing small, medium, and large respectively.

Metric	Predictor/Effect	Corrected p-value	Effect Size (η^2^)	Interpretation
Stroke Volume	Exercise	<0.0000	0.190	Exercise is a significant predictor of SV showing a very large effect.
Stroke Volume	Sex	<0.0000	0.127	Sex is a significant predictor of SV showing a medium to large effect.
Stroke Volume	Genotype	0.0491	0.044	Exercise is a significant predictor of SV showing a small to medium effect.
Stroke Volume	Exercise:Sex	0.0479	0.034	The interaction of exercise and sex has a significant, small to medium effect on SV.
Cardiac Output	Exercise	<0.0000	0.194	Exercise is a significant predictor of CO showing a very large effect.
Cardiac Output	Sex	0.0001	0.109	Sex is a significant predictor of CO showing a very medium to large effect.
Cardiac Output	Exercise:Sex	0.0491	0.035	The interaction of exercise and sex has a significant, small to medium effect on CO.
Cardiac Index	Exercise	<0.0000	0.197	Exercise is a significant predictor of CI showing a very large effect.
Cardiac Index	Genotype	0.0479	0.057	Genotype is a significant predictor of CI showing a medium effect.
RV Stroke Volume	Exercise	<0.0000	0.148	Exercise is a significant predictor of RVSV showing a large effect.
RV Stroke Volume	Sex	<0.0000	0.120	Sex is a significant predictor of RVSV showing a medium to large effect.
RV Stroke Volume	Sex:HN	0.0127	0.052	The interaction of sex and HN factor has a significant, small to medium effect on CO.
RV Ejection Fraction	Exercise	0.0003	0.116	Exercise is a significant predictor of RVEF showing a medium to large effect.
RV Ejection Fraction	Sex	0.0491	0.045	Sex is a significant predictor of RVEF showing a small to medium effect.

### Stratified subgroup exercise comparison results

**Fig 4:**
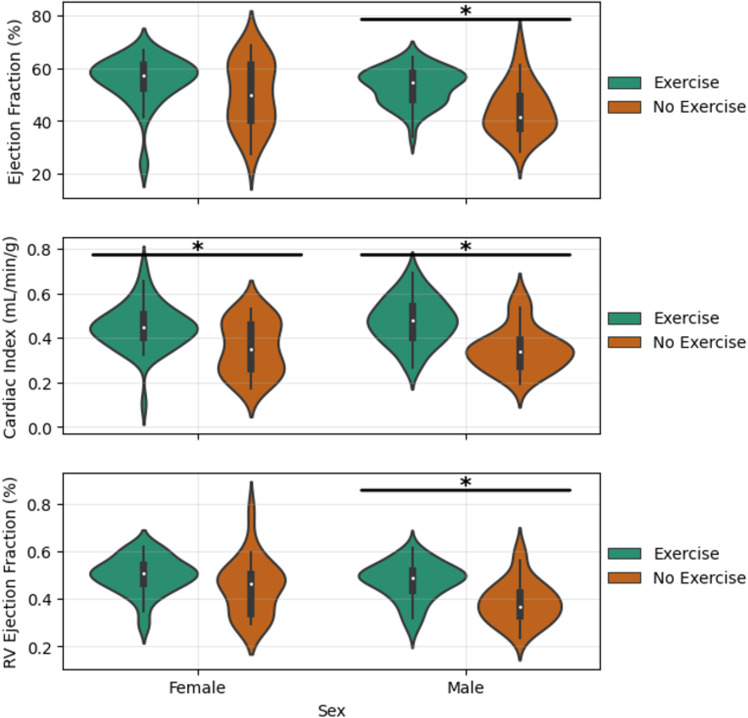
Collection of violin plots showing the differences between exercised and nonexercised mice subdivided by sex. The bars with asterisks indicate statistically significant differences revealed by a Mann Whitney U test (P<0.05).

**Fig 5:**
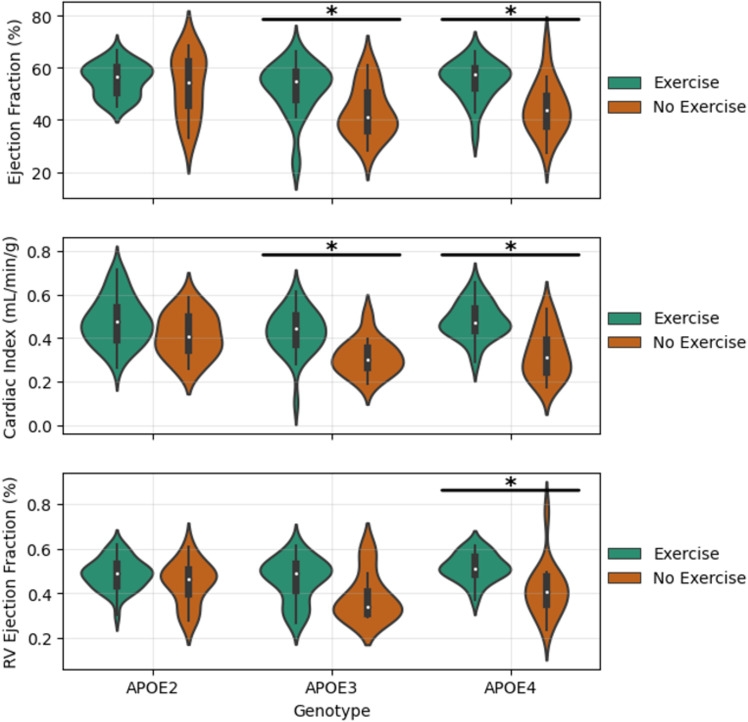
Collection of violin plots showing the differences between exercised and nonexercised mice subdivided by.

Within our stratified analysis of exercise group differences, we focused only on EF, CI, and RVEF. EF is a normalized measure that accounts for baseline differences in heart size. CI is a normalized measure that accounts for differences in body mass. These normalized metrics were chosen to reduce the impact of the mass differences (Fig 3) which are not exercise related. Figs 4 and 5 contain key violin plots demonstrating the stratified group differences our study revealed between exercised and nonexercised mice. Table 6 gives a summary of all significant stratified group differences. Across the board, exercise has a marked positive impact on EF, CI, and RVEF. This improvement is especially pronounced in males, as indicated in Fig 4, where both EF and RVEF are significantly higher in exercised males compared to their non-exercised counterparts (p < 0.0007 for EF and RVEF, Cohen’s D of 1.16 and 1.08, respectively). The effect is also observed in females, but to a lesser degree, with significant improvement in CI (p = 0.0142, Cohen’s D of 0.89). Fig 5 highlights that the response to exercise is genotype dependent. APOE3 and APOE4 mice show significant improvements in EF, CI, and RVEF with exercise. In particular, the APOE4 group shows the most robust response, with EF increasing significantly (p = 0.0028, Cohen’s D of 1.27) and CI showing the largest effect size (Cohen’s D of 1.64). Conversely, the APOE2 group exhibits no significant improvements with exercise, indicating a less responsive phenotype to exercise interventions in this genotype. The large effect sizes (Cohen’s D ranging from 0.89 to 1.64) reported for different metrics and subgroups highlight the importance of exercise in enhancing cardiac function, particularly in the APOE3 and APOE4 genotypes and in males.

**Table 6:** A summary of the significant differences (p<0.05) identified by the stratified analysis of exercise within sex and genotype subgroups.

Metric	Stratified Subgroup for Comparison	Corrected p-value	Cohen’s D	Interpretation
Ejection Fraction	Males Only	0.0007	1.16	Within males, there is a significant difference in EF between mice with and without exercise.
Cardiac Index	Females Only	0.0142	0.89	Within females, there is a significant difference in CI between mice with and without exercise.
Cardiac Index	Males Only	0.0003	1.26	Within males, there is a significant difference in CI between mice with and without exercise.
RV Ejection Fraction	Males Only	0.0007	1.08	Within males, there is a significant difference in RVEF between mice with and without exercise.
Ejection Fraction	APOE3 Only	0.0087	0.94	Within mice with the APOE3 allele, there is a significant difference in EF between those with and without exercise.
Ejection Fraction	APOE4 Only	0.0028	1.27	Within mice with the APOE4 allele, there is a significant difference in EF between those with and without exercise.
Cardiac Index	APOE3 Only	0.0018	1.23	Within mice with the APOE3 allele, there is a significant difference in CI between those with and without exercise.
Cardiac Index	APOE4 Only	0.0007	1.64	Within mice with the APOE4 allele, there is a significant difference in CI between those with and without exercise.
RV Ejection Fraction	APOE4 Only	0.0007	1.18	Within mice with the APOE4 allele, there is a significant difference in RVEF between those with and without exercise.

genotype. The bars with asterisks indicate statistically significant differences revealed by a Mann Whitney U test.

## Discussion

Our results confirm that automated segmentation allows for fast and accurate quantitative measurements. Qualitative assessment of the segmentations provides added confidence in the accuracy of this work. The segmentation performance (as shown in Table 3) using CT images as input generally outperformed the results when using I maps. This confirms previous findings that segmentation with CT input offers higher accuracy and overlap metrics, such as the Dice and Jaccard coefficients, reflecting clearer tissue boundaries [12]. In contrast, segmentation with I maps as input shows a notable decline in these metrics, reflecting the challenge of segmenting images with enhanced noise or less distinct tissue boundaries. Precision remains high for both inputs, indicating that the model avoids false positives, but recall is somewhat lower, suggesting it misses some boundaries, especially with I maps.

The multi-factor statistical analyses highlight clear differences in cardiac performance in our cohort. For nearly all cardiac functional metrics, exercise was revealed to be a significant predictor with a large effect size (Table 5). This confirms the beneficial influence of exercise on cardiac performance across the cohort. In addition to exercise, sex and genotype were also significant factors, although their effects were smaller. Notably, the interaction between exercise and sex suggests that males benefit more from exercise, as supported by the stratified subgroup analysis. This interaction aligns with the stronger exercise-induced improvements in males observed for EF, CI, and RVEF, as shown in Figs 4 and 5.

Interestingly, the results reveal no significant impact of exercise on overall mass, as demonstrated by the Kruskal-Wallis and Dunn’s post hoc analyses in Fig 3. Mass differences in our cohort are driven primarily by sex and genotype, rather than by exercise. This reinforces the idea that exercise predominantly affects cardiac function rather than structural metrics like heart mass. The analysis also revealed an interaction between sex and the HN factor influencing right ventricular stroke volume, suggesting that the humanized innate immune component might modulate sex-specific responses to cardiovascular stimuli like exercise.

The stratified group comparisons showed that exercise significantly enhances cardiac metrics, including EF, CI, and RVEF. Male mice generally exhibit greater improvements than females (as seen in Fig 4), reflecting sex-specific responses likely driven by physiological factors such as hormonal differences, as males typically exhibit a greater capacity for exercise-induced cardiac hypertrophy and functional improvement. Furthermore, cardiac function of APOE2 genotypes was unaffected by exercise while both APOE3 and APOE4 mice showed a significant improvement with exercise (Fig 5). These findings suggest that the APOE4 genotype is particularly responsive to exercise, consistent with previous studies [33,34] showing that the increased disease risk in this genotype can be mitigated by exercise.

These findings underscore the importance of personalized approaches when considering exercise as a therapeutic intervention, especially for cardiovascular conditions. The variability in responses, as indicated by the standard deviations, suggests underlying biological mechanisms that may differ across individuals, warranting further exploration. Exercise stands out as a strong determinant of cardiac performance, particularly for male mice with APOE3 and APOE4 genotypes, reinforcing the potential of targeted exercise regimens for those with genetic predispositions. Differences between genotypes and sexes also provide practical insights for lifestyle interventions tailored to specific genetic backgrounds, particularly in males, where exercise consistently improves multiple cardiac metrics, while female benefits are genotype restricted. Still, within sex differences indicate that APOE4 females benefit more than APOE3 and APOE2 females.

The observed differences in exercise benefits between male and female mice can likely be explained by the distinct biological effects of sex hormones on cardiovascular function. Estrogen, which is more prevalent in females, offers cardioprotective effects such as improved endothelial function, anti-inflammatory properties, and enhanced lipid metabolism. This protective baseline could reduce the additional benefits of exercise, as female cardiovascular health is already supported by hormonal protection, particularly premenopausal females with higher estrogen levels [35,36]. In males, testosterone promotes cardiac hypertrophy and enhanced cardiovascular performance, which could amplify the effects of exercise. Additionally, APOE4 males typically exhibit a more pro-inflammatory and pro-atherogenic profile, which exercise helps to improve, aligning with previous findings on the cardiovascular risks associated with the APOE4 genotype [2]. These results are consistent with the broader understanding that sex-specific responses to exercise are complex and heavily influenced by genetic background.

In our previous study [12], we found that high-fat diet negatively affects cardiac performance, especially in APOE4 and APOE2 genotypes, leading to increased left ventricular volumes and reduced ejection fractions. In contrast, the current study demonstrates that exercise significantly enhances cardiac function, particularly in male APOE3 and APOE4 mice, suggesting that exercise serves as a robust cardioprotective intervention. The findings highlight the contrasting impacts of high-fat diet and exercise, underscoring the importance of lifestyle modifications in managing genetic predispositions to cardiovascular disease. Specifically, exercise not only mitigates the cardiovascular risks associated with APOE genotypes, but also offers a targeted intervention to improve cardiac function in susceptible populations. While exercise was evaluated independent of diet in this work, future work may include joint evaluation of diet and exercise.

## Limitations

While this study demonstrates significant findings regarding the cardioprotective effects of exercise across APOE genotypes using photon-counting CT and deep learning, several limitations should be acknowledged:

1.** Sample Size and Subgroup Variability:** Although the study includes a relatively large cohort of 140 mice, the sample sizes for some subgroups (e.g., genotype and sex combinations) are limited, particularly for non-exercised mice. This may have reduced the statistical power of some analyses, especially for less responsive genotypes such as APOE2.
2.** Cross-Sectional Design:** This study examines the effects of exercise at a single time point. A longitudinal design would allow us to evaluate how cardiac function evolves with exercise over time, providing deeper insights into the dynamics of exercise-induced adaptations.
3.** Potential Bias in Deep Learning Segmentation:** Although the deep learning model achieved high accuracy and Dice coefficients, the training and validation datasets were based on semi-automatically labeled data from a subset of mice. This may introduce labeling biases that could influence the segmentation performance, especially in complex cardiac structures.
4.** Absence of Functional Behavior Assessment:** While cardiac metrics were rigorously quantified, the study did not include functional assessments such as behavioral or endurance testing to link cardiac improvements to broader physiological or performance outcomes.
5.** No Exploration of Molecular Mechanisms:** The study provides a phenotypic analysis of cardiac function but does not explore the molecular or cellular mechanisms underlying the observed genotype- and sex-specific responses to exercise. Investigating these mechanisms could offer valuable insights into the biological pathways affected by exercise.
6.** Clinical Translation:** While this study demonstrates the utility of photon-counting CT in preclinical research, direct translation of the findings to human studies may be challenging due to species-specific differences in cardiac physiology and APOE-related effects.

These limitations highlight areas for improvement in future studies, such as incorporating longitudinal designs, expanding subgroup sizes, and exploring molecular mechanisms. Despite these constraints, the study provides valuable insights into genotype- and sex-specific exercise responses and underscores the utility of advanced imaging technologies in preclinical cardiovascular research.

## Conclusion

These findings underscore the complex interplay between APOE genotype, sex, and exercise in modulating cardiac function. The significant improvements in cardiac metrics due to exercise, particularly in male and female APOE3 and APOE4 mice, suggest that targeted lifestyle interventions could have differential benefits depending on sex and genetic background, potentially mitigating cardiovascular risks associated with specific APOE genotypes.

## Supporting information

S1 Table 1 Supplemental table showing all cardiac chamber volumes. Averages and standard deviations for measured cardiac volumes grouped by sex, genotype, and exercise state. Standard deviations are shown in parentheses below each average value. From left to right, the measurements are of the diastolic left ventricle (DLV), systolic left ventricle (SLV), diastolic right ventricle (DRV), systolic right ventricle (SRV), diastolic left atrium (DLA), systolic left atrium (SLA), diastolic right atrium (DRA), and systolic left atrium (SRA). (DOCX)
