# Supplementary material for: Assessing the cardioprotective effects of exercise in APOE mouse models using deep learning and photon-counting micro-CT
Source: PLoS One. 2025 Apr 10;20(4):e0320892. doi: 10.1371/journal.pone.0320892 (PMC11984728; doi:10.1371/journal.pone.0320892)
Supplement: S1 Table 1 — (DOCX) [file pone.0320892.s001.docx]

**S1 Table 1. Supplemental table showing all cardiac chamber volumes**. Averages and standard deviations for measured cardiac volumes grouped by sex, genotype, and exercise state. Standard deviations are shown in parentheses below each average value. From left to right, the measurements are of the diastolic left ventricle (DLV), systolic left ventricle (SLV), diastolic right ventricle (DRV), systolic right ventricle (SRV), diastolic left atrium (DLA), systolic left atrium (SLA), diastolic right atrium (DRA), and systolic left atrium (SRA).

| **Sex** | **Genotype** | **Exercise?** | **DLV**  **(mL)** | **SLV**  **(mL)** | **DRV**  **(mL)** | **SRV**  **(mL)** | **DLA**  **(mL)** | **SLA**  **(mL)** | **DRA**  **(mL)** | **SRA**  **(mL)** |
| --- | --- | --- | --- | --- | --- | --- | --- | --- | --- | --- |
| Male | APOE2 | Yes | 0.060  (0.016) | 0.027  (0.010) | 0.063  (0.017) | 0.033  (0.013) | 0.017  (0.007) | 0.021  (0.006) | 0.021  (0.013) | 0.030  (0.011) |
|  |  | No | 0.055  (0.007) | 0.029  (0.008) | 0.061  (0.012) | 0.036  (0.012) | 0.020  (0.005) | 0.021  (0.005) | 0.026  (0.010) | 0.033  (0.008) |
|  | APOE3 | Yes | 0.064  (0.012) | 0.031  (0.009) | 0.069  (0.016) | 0.039  (0.014) | 0.012  (0.004) | 0.020  (0.005) | 0.013  (0.007) | 0.028  (0.007) |
|  |  | No | 0.060  (0.008) | 0.037  (0.010) | 0.061  (0.008) | 0.040  (0.008) | 0.013  (0.003) | 0.018  (0.001) | 0.020  (0.012) | 0.030  (0.008) |
|  | APOE4 | Yes | 0.064  (0.019) | 0.031  (0.015) | 0.058  (0.008) | 0.029  (0.006) | 0.017  (0.006) | 0.024  (0.006) | 0.016  (0.007) | 0.028  (0.006) |
|  |  | No | 0.045  (0.005) | 0.025  (0.005) | 0.047  (0.008) | 0.029  (0.006) | 0.015  (0.003) | 0.018  (0.002) | 0.016  (0.006) | 0.023  (0.004) |
| Female | APOE2 | Yes | 0.050  (0.008) | 0.022  (0.005) | 0.050  (0.006) | 0.025  (0.005) | 0.013  (0.004) | 0.020  (0.003) | 0.010  (0.005) | 0.022  (0.005) |
|  |  | No | 0.042  (0.006) | 0.016  (0.003) | 0.047  (0.011) | 0.024  (0.006) | 0.007  (0.002) | 0.014  (0.003) | 0.011  (0.006) | 0.024  (0.010) |
|  | APOE3 | Yes | 0.047  (0.014) | 0.023  (0.013) | 0.047  (0.010) | 0.024  (0.005) | 0.010  (0.007) | 0.016  (0.005) | 0.010  (0.006) | 0.020  (0.006) |
|  |  | No | 0.045  (0.001) | 0.024  (0.004) | 0.046  (0.005) | 0.028  (0.008) | 0.010  (0.003) | 0.016  (0.001) | 0.010  (0.005) | 0.020  (0.004) |
|  | APOE4 | Yes | 0.041  (0.004) | 0.017  (0.004) | 0.040  (0.003) | 0.019  (0.003) | 0.010  (0.002) | 0.016  (0.002) | 0.008  (0.004) | 0.020  (0.003) |
|  |  | No | 0.040  (0.007) | 0.023  (0.007) | 0.041  (0.010) | 0.023  (0.009) | 0.012  (0.003) | 0.016  (0.003) | 0.014  (0.005) | 0.025  (0.006) |
